# Postoperative opioid-free analgesia in dogs undergoing tibial plateau leveling osteotomy: a feasibility study

**DOI:** 10.3389/fvets.2024.1394366

**Published:** 2024-07-05

**Authors:** Caroline Didier, Sarah Faucher, Marti Sarra Ferrer, Mathilde Lapouge, Stéphane Junot, Géraldine Jourdan

**Affiliations:** ^1^Department of Clinical Sciences, National Veterinary School of Toulouse, University of Toulouse, Toulouse, France; ^2^Anicura Medicabanis Veterinary Hospital, L’Union, Lyon, France; ^3^Department of Veterinary Anesthesia and Analgesia, Université de Lyon, VetAgro Sup, Marcy l’Etoile, France; ^4^RESTORE Research Center, University of Toulouse, INSERM, CNRS, EFS, ENVT, Toulouse, France

**Keywords:** analgesia, loco-regional, opioid-free anesthesia, dog, welfare, postoperative pain, TPLO

## Abstract

**Objectives:**

This study was designed to prospectively evaluate the feasibility of an opioid-free anesthesia protocol and describe the quality of recovery and management of postoperative analgesia in dogs after a tibial plateau leveling osteotomy (TPLO).

**Methods:**

In total, 20 dogs presented for TPLO were included. After premedication with intravenous (IV) medetomidine (0.005–0.007 mg/kg) and midazolam (0.2 mg/kg), the dogs were anesthetized using ketamine (2 mg/kg) and propofol and maintained with isoflurane and ketamine CRI (0.6 mg/kg/h). Sciatic and femoral nerve blocks were performed with bupivacaine 0.5% (0.087 +/− 0.01 and 0.09 +/− 0.02 mL/kg, respectively). Meloxicam (0.2 mg/kg IV) was administered intraoperatively, after osteotomy. Fentanyl (0.002 mg/kg IV) was administered intraoperatively, as rescue analgesia in the case of sustained increase in cardiorespiratory variables. Two pain scores (French 4A-VET and Glasgow short form) were performed at conscious sternal recumbency and 2, 4, 6, 8, 12, and 20 h after extubation and compared to baseline using a Friedman test followed by a Nemenyi *post-hoc* test. The time taken for the first food intake and urination was reported.

**Results:**

Intraoperative opioid-free anesthesia was feasible in 11 dogs, whereas 9 dogs received fentanyl once during arthrotomy. No opioid postoperative rescue analgesia was required. Food intake occurred within 6 h, and all dogs were discharged after 24 h without any complication.

**Conclusion:**

Total opioid-free postoperative analgesia was achieved in all dogs, with adequate recoveries. Although opioid-free anesthesia was feasible in 55% of the population, a single dose of fentanyl was necessary in 45% of the dogs during arthrotomy.

## 1 Introduction

Opioids have represented the core of perioperative analgesia in humans and veterinary medicine for decades (1). Over the past few years, major side effects associated with their widespread use have been well characterized in people including respiratory depression, gastrointestinal disorders, urinary retention, and postoperative delirium (1, 2). Hyperalgesia and tolerance phenomenon, commonly observed in humans, lead to increased opioid consumption and potential addiction, which triggered the major crisis currently in North America and Europe, and caused a paradigm shift in perioperative analgesic management tending to decrease opioid usage (1, 3). Most of these side effects are also described in veterinary medicine (4) including withdrawal syndrome in laboratory animals (5, 6). If the latter is hardly described in dogs, the benefits of opioid restriction on postoperative general behavior have been reported in some studies, similar to human medicine. For example, Bini et al. reported that lower opioid consumption was associated with better demeanor and faster return to normal nutritional behavior after tibial plateau leveling osteotomy (TPLO) in dogs that received a pain score-based conservative analgesia compared to those administered a systematic opioid regimen every 4 h (7). In addition, Palomba et al. reported a faster return to food intake associated with reduced doses of postoperative methadone in dogs that received a peripheral nerve block for TPLO compared to intraoperative fentanyl-based systemic analgesia (8). This supports the potential benefits of reducing postoperative opioid use for the well-being our veterinary patients.

Opioid-sparing anesthesia (OSA) is defined as a technique where small amounts of opioids may be used intraoperatively (single-dose and/or low-dose opioids) (9), ultimately culminates in opioid-free anesthesia (OFA) when total avoidance of opioids can be achieved (3). Both practices have been implemented and documented in people (10–12): it usually consists of a combination of NSAIDs with acetaminophen or nefopam, often associated with local analgesia and continuous infusions of ketamine, dexmedetomidine, and less frequently lidocaine or magnesium (1). Over the past few years, veterinary medicine followed the tracks with several case reports (13–16) and a few original studies (17–21). More recently, several veterinary studies evaluated the analgesic efficacy of OSA and OFA protocols specifically on perioperative nociception and pain in stifle surgeries (22–28). These studies have reported either intraoperative total avoidance (25, 26) or a significant postoperative decrease (23, 28) of opioid consumption. However, significant benefits of total OFA over opioid-based protocols remain difficult to prove against potential adverse events reported, especially when constant rate infusions (CRI) of non-opioid drugs are used without locoregional techniques (29). Despite its several advantages, OFA remains still inconsistently used (12, 30). In addition, to the best of our knowledge, total opioid-free postoperative analgesia (tOF-poA) following OFA protocols has never been reported in the veterinary literature.

Our objectives were to investigate the feasibility of an anesthetic protocol designed as OFA for the management of intra- and postoperative analgesia and to describe the quality of recovery of dogs presented for TPLO. We hypothesized that our OFA protocol would cover intraoperative nociception, with the potential use of a single-dose bolus of fentanyl as rescue analgesia (OSA), but also fully address postoperative pain resulting in total avoidance of opioids with an optimal patient’s comfort until discharge.

## 2 Materials and methods

This study was designed as a prospective clinical observational trial. The study design was approved by the University Institutional Animal Care and Use Committee of Toulouse University (SSA_2022_013).

### 2.1 Animals

Dogs presented to the University Teaching Hospital of the National Veterinary School of Toulouse between September and December 2022 and scheduled for elective TPLO were assessed for eligibility. After physical examination, serum biochemistry, and complete blood count, healthy dogs identified with an American Society of Anesthesiologists (ASA) physical status ≤ 2 were includedin the study. Dogs receiving oral opioids, presenting with concomitant osteoarticular injury or any contraindication to the regular use of alpha-2 adrenoreceptors agonists, were excluded. Any non-opioid analgesic drug administration during the week prior to surgery was recorded.

### 2.2 OFA protocol

Dogs were fasted 8 h before being admitted to the hospital on the morning of surgery. Weight and body condition scores were recorded as part of the pre-anesthetic physical examination. An anxiety score (0–16) (31), a sedation score (a simple descriptive scale used at our institution: 0 = no sedation, 1 = minor sedation, 2 = moderate sedation, and 3 = deep sedation), and two pain scores [The French 4A-VET canine acute pain scale (4A-VET-CAPS) and the Glasgow composite measure pain scale—short form (GCMPS-SF)] were performed after a minimal 30-min acclimation period in wards_._ These were considered baseline values.

A cephalic vein was cannulated for intravenous (IV) premedication with medetomidine (Domitor; Orion Pharma, France) either at 0.005 or 0.007 mg/kg for dogs weighing more or less than 25 kg, respectively, followed by midazolam 0.2 mg/kg (Midazolam; Viatris, France) 5 min later; and after 2 moderate, sedation was scored. The dog was pre-oxygenated for 3 min before induction with 2 mg/kg of IV ketamine (Ketamine 1,000; Virbac, France) and propofol (Propovet; Abbott, France) titrated to allow endotracheal intubation. Anesthesia was maintained with isoflurane (Isoflow; Abbott, France) in 100% oxygen. A ketamine CRI was started at 0.6 mg/kg/h and continued throughout anesthesia. The end-expiratory isoflurane fraction was maintained between 1.3 and 1.4% during the entire procedure (Datex Ohmeda S/5 Anesthesia Monitor; General Electrics, Finland). Lactated Ringer’s solution was administered at a rate of 5 mL/kg/h (Ringer Lactate; B. Braun, France). Monitoring included clinical assessment of anesthesia depth, heart rate (HR), respiratory rate (RR), ECG, pulse oximetry (SpO_2_), capnography, non-invasive blood pressure (NIBP), and esophageal temperature (T) (Datex Ohmeda S/5 Anesthesia Monitor; General Electrics, Finland). The dorsal pedal artery of the non-operated pelvic limb was cannulated before transfer to the operating theater for subsequent invasive blood pressure (IBP) monitoring.

After aseptic preparation of the operated pelvic limb, femoral and sciatic nerve blocks were performed under ultrasound guidance (Mylab^™^ One; Esaote, Italy), completed with low-intensity neurostimulation (Stimuplex HNS 12; B. Braun Medical, France) by a single operator (CD). The sciatic and femoral nerve blocks were performed with a 13-MHz linear array ultrasound transducer positioned immediately distal to the trochanter major and at the level of the femoral triangle, respectively, as previously described (32). For each block, an insulated needle (Stimuplex Insulated Needle; B. Braun, France) of appropriate length was advanced in plane until penetration of the fasciae surrounding the nerves. An absence of motor response to a stimulation current of 0.2 mA ensured sufficient nerve-to-needle distance, and adequate positioning was confirmed by observation of a “donut sign” around the target nerves upon the administration of a 0.5 mL test volume of bupivacaine 0.5% (Bupivacaïne, Aguettant, France). The total volume of bupivacaine (0.05 to 0.1 mL/kg) administered per nerve was chosen based on the operator’s appreciation of nerve-to-needle distance and hydrodissection of fasciae.

If no major bleeding, hypothermia (T < 35°C), or hypotension meloxicam0.2 mg/kg IV (Metacam; Boehringer, France) and acepromazine (Calmivet; Vetoquinol, France) were administered intraoperatively, immediately after osteotomy. The dosage of acepromazine was chosen based on the admission anxiety score, 0.005 or 0.01 mg/kg IV for patients scored ≤ 4 or > 4 respectively.

### 2.3 Intraoperative management and nociception evaluation

The same orthopedic surgeon (ML) performed each procedure, starting with an arthrotomy to evaluate the extent of the rupture of the cranial cruciate ligament before the osteotomy phase.

Physiological variables were recorded every 5 min during anesthesia. Intraoperative nociception was assessed by two anesthetists (CD and SF) and defined as a 5-min-long, 20% increase, of at least two of the following: HR, mean IBP (MAP), and RR associated with surgical stimulation, in the absence of any other confounding circumstances (e.g., emergence, major bleeding, and hyperthermia), compared to the average values of the last 10 min (25). For ethical reasons, fentanyl of 0.002 mg/kg IV (Fentadon; Dechra, France) would then be administered as rescue analgesia and the protocol subsequently requalified as OSA (9). If more than two boluses were needed to control sustained nociception, a CRI would be initiated at 2 μg/kg/h. This would be considered a block failure, and the protocol would be requalified as opioid-based with fentanyl CRI. Duration of surgery and anesthesia, time from the end of anesthesia to tracheal extubation, and time from tracheal extubation to conscious sternal recumbency were recorded. The recovery quality was evaluated during the latter period and scored as standard practice at our institution: 1 = calm, quiet; 2 = minor pain and/or minor excitement; 3 = moderate pain and/or excitement; and 4 = severe pain and/or excitement.

### 2.4 Postoperative management

After achieving conscious sternal recumbency, ketamine CRI was decreased from 0.6 to 0.3 mg/kg/h for 6 h, followed by 0.18 mg/kg/h for 14 h before discontinuation. The pain was evaluated using two pain scales (4A-VET-CAPS and GCMPS-SF) at the time of conscious sternal recumbency, and 2, 4, 6, 8, 12, and 20 h after tracheal extubation. Methadone was administered as rescue analgesia, 0.1 or 0.2 mg/kg IV, when at least 1 of the 2 pain scores reached 5 or 10, respectively. At each time point until 8 h after tracheal extubation, a sedation score was recorded. Motor blockade was assessed at any time point. It was considered present when proprioception testing was abnormal (absent or retarded reposition of the leg). Immediate repositioning of the leg indicated an absence of motor blockade. A small amount of food was offered at each time point starting 2 h after tracheal extubation. The time to first food intake and urination was recorded for each dog.

### 2.5 Statistical analysis

Statistical analysis was performed as simple descriptive statistics using commercially available RealStats-Mac-2016^®^ commercial software. Data were assessed for normality using a Shapiro–Wilk test. Normally distributed data are summarized as mean +/− standard deviation and non-normally distributed ones are presented as median [range]. Pain and sedation scores were compared among all the relevant time points and to baseline using a Friedman non-parametric ANOVA for repeated measures followed when required by a Nemenyi *post-hoc* test with Bonferroni correction. A *p*-value of less than 0.05 was considered significant.

## 3 Results

### 3.1 Animals

During the study period, 20 dogs met the inclusion criteria and were assessed for eligibility. Of these, 16 females (15 spayed and 1 intact) and 3 males (1 neutered and 2 intact) were enrolled. Breeds were distributed as follows: mixed breed dogs, seven; Golden Retrievers, three; Cane Corso, two; and one each of Yorkshire Terrier, German Shorthaired Pointer, American Staffordshire Terrier, Setter Gordon, Boxer, Shih Tzu, French Mastiff, and Brittany Spaniel.

The median age and weight were 5.1 [1.3–11.8] years and 30 [7.1–50] kg, respectively, with a median BCS of 5 out of 9 [4–8]; 1 dog was known to have an MDR1 heterozygous positive mutation, and another one had a previous history of gastrointestinal side effects when given meloxicam. Acepromazine for the first one and meloxicam for the second one were removed from their protocol. The latter was replaced with postoperative oral acetaminophen. Pre-anesthetic blood tests were unremarkable in all dogs.

The median anxiety score was 4 [2–10].

### 3.2 Intraoperative variables

#### 3.2.1 Femoral and sciatic nerve blocks

Ultrasound guidance was achieved for all sciatic and 18 femoral blocks. Due to vascular puncture, two femoral blocks were eventually performed with electro-location only via a pre-iliac approach. The actual mean volumes of bupivacaine injected at the level of the sciatic and femoral nerves were 0.087 +/− 0.01 (range [0.059–0.01]) and 0.09 +/− 0.02 mL/kg ([0.062–0.011]), respectively.

#### 3.2.2 Anesthesia

The mean duration of anesthesia and surgery was 241 +/− 27 min and 111 +/− 17 min, respectively. Successful arterial cannulation allowed IBP monitoring in 16 dogs, whereas 4 had only NIBP monitored. End-tidal carbon dioxide was maintained between [35–45] mmHg, and SpO_2_ was stable above 97%. No dog experienced hypotension during anesthesia. The only reported complications were transient and light emergence phases only during transfers to the operating theater, preparation room, or radiology, which were managed with the administration of propofol (0.5 to 1 mg/kg IV) or increased isoflurane.

In total, 19 dogs received meloxicam immediately after osteotomy, and 1 dog was administered 15 mg/kg of acetaminophen (Doliprane; Sanofi, France) orally twice for the first 24-h postoperative period. Acepromazine was administered IV at 0.01 mg/kg and 0.005 mg/kg to 8 and 11 dogs based on anxiety scores, more than 120 min after premedication with medetomidine. Times from the end of anesthesia to tracheal extubation and from tracheal extubation to conscious sternal recumbency were 9.7 +/− 4.6 min and 15.8 +/− 10.4 min, respectively. The median recovery quality score and median sedation score at conscious sternal recumbency were 1 [1–2] and 1[1–2], respectively. Overall, all recoveries were calm and quiet, and conscious sternal recumbency occurred within 30 min from tracheal extubation, allowing early postoperative pain assessment in all dogs.

#### 3.2.3 Rescue analgesia

In total, 9 dogs required a single bolus of fentanyl when the arthrotomy was performed, 81.8 +/− 13.9 min after the nerve block. No other rescue analgesia was needed intraoperatively in any patient. Therefore, 11 dogs received an intraoperative OFA protocol, whereas in 9 dogs, the protocol was converted to intraoperative OSA. No block failure was reported.

### 3.3 Postoperative variables

Pain scores for 4A-VET-CAPS were ≤ 4 [0–18] at each time point for all patients. Pain scores for GCMPS-SF were ≤ 4 [0–24] at each time point for all patients. No dogs required rescue opioid analgesia in the 24 h postoperative period. Detailed results for 4A-VET-CAPS and GCMPS-SF pain scores are presented in Table 1, and no score was statistically different postoperatively compared to baseline. Sedation scores were significantly higher than baseline only after premedication and at the sternal recumbency time point. The first food intake occurred in 19 out of 20 dogs during the 6 h following tracheal extubation. The remaining dog ate only 16 h after extubation, with his owner. This dog had an anxiety score of 7 out of 16. Motor blockade remained present in all dogs 6 h after tracheal extubation and had disappeared at the time of the last evaluation (20 h after extubation) in all dogs except one. Return to normal proprioception in the last dog occurred before discharge but outside the 20-h evaluation period and was not precisely recorded. In total, 15 dogs spontaneously urinated 8 h after extubation, 4 overnight, and 1 the next morning. All were discharged the day after surgery without complications.

**Table 1:** Sedation and pain scores in 20 dogs upon admission (baseline), after premedication (post-PM), at conscious sternal recumbency (SR) and 2, 4, 6, 8, 12, and 20 h following tracheal extubation.

Variable	Time points (*H* = *hours after extubation*)								
	*Baseline*	*Post-PM*	*SR*	*H = 2*	*H = 4*	*H = 6*	*H = 8*	*H = 12*	*H = 20*
Sedation (0–3)	0	2 [1–3]*	1 [1–2]*	1 [0–1]	1 [0–1]	0 [0–0]	0 [0–0]	/	/
4A-VET-CAPS (0–18)	1 [0–2]	/	2 [0–4]	2 [1–3]	1 [1–3]	1 [1–4]	1 [1–4]	1 [0–3]	1 [0–2]
GCMPS-SF (0–24)	1 [0–4]	/	1 [1–3]	1 [2–3]	1 [1–3]	1 [0–2]	1 [0–4]	1 [0–3]	1 [0–4]

Pain scoring systems were the Glasgow composite measure pain scale—short form (GCMPS-SF) and 4A-VET canine acute pain scale (4A-VET-CAPS). Sedation scores are evaluated based on a simple descriptive scale: 0 = no sedation, 1 = minor sedation, 2 = mild sedation, and 3 = deep sedation. Data are presented as median [min–max]. / variable not evaluated at the considered time point. *statistically significant difference from baseline (*p* < 0.05).

## 4 Discussion

This study describes a multimodal analgesia approach including femoral and sciatic (FS) blocks to achieve total opioid-free postoperative analgesia (tOF-poA) in 20 dogs undergoing a TPLO. During the intraoperative period, 55% of the dogs received an OFA protocol, whereas 45% were successfully managed with OSA (9). Calm and quiet recoveries were followed by uneventful early postoperative convalescence. Moreover, no block failure was reported in our cohort.

Opioid-free analgesia involves the combination of various opioid-sparing strategies culminating in complete avoidance of perioperative opioid usage to reduce subsequent adverse events without sacrificing the patient’s comfort (3). The aim is to reduce opioid-induced bradycardia and hypoventilation, postoperative nausea, gastroesophageal reflux and vomiting, dysphoria, and supposedly postoperative hyperalgesia (1). In human medicine, it usually consists of a combination of NSAIDs with acetaminophen or nefopam, often associated with local analgesia and continuous infusions of ketamine, dexmedetomidine, and less frequently lidocaine or magnesium (1). The use of gabapentin in association with NSAIDs also successfully reduced opioid consumption in humans (33). Similar to human protocols, our cohort benefited from immediate postoperative analgesia with NSAIDs associated with a ketamine CRI. Both were started during the intraoperative period to initiate early anti-inflammatory pain management. The one dog that was intolerant to NSAIDs received oral acetaminophen instead in the early postoperative period. Meloxicam and acetaminophen inhibit action of inflammatory cyclo-oxygenase and alleviate the severe inflammation accompanying such invasive procedures (34). When left uncontrolled, inflammation is actively involved in cytokine storms and persistent nociceptors activation that promote peripheral sensitization, eventually leading to increased pain perception. Hence, NSAIDs and acetaminophen used in multimodal analgesia protocols have often been reported to decrease opioid consumption in the postoperative period (33). As an N-methyl-D-aspartate (NMDA) antagonist, ketamine administered as a low-dose CRI is primarily used in our protocol for its anti-proinflammatory properties and to prevent perioperative hyperalgesia. It blocks glutamate release and decreases excitatory inputs at the synaptic junction between nociceptors and interneurons in the dorsal horn of the spinal cord. The interest in its perioperative use at low dosages for analgesic properties has been renewed, especially in OSA/OFA protocols (33). First, its NMDA-receptor antagonism has been proposed to counteract different levels of inflammation such as recruitment of inflammatory cells, production of cytokines, and regulation of inflammatory mediators, conferring to ketamine, an anti-proinflammatory effect by limiting exacerbation of systemic inflammation (35). Second, as both opioid-induced and surgical hyperalgesia are largely due to a dose-dependent NMDA receptor activation, ketamine-induced NMDA antagonism has been reported to decrease both postoperative pain intensity and general opioid consumption and significantly improve postoperative analgesia in several meta-analyses in human medicine (36, 37). The opioid-sparing effect of ketamine CRI alone has never been proven in veterinary medicine, but some studies showed improvement in general demeanor, feeding behavior, and analgesia quality during the postoperative period (38, 39). Similarly, in the present cohort, overall postoperative pain management and comfort were deemed very satisfactory. Additionally, the preventive use of acepromazine in all dogs provided light tranquilization and anxiolysis for recovery, when any residual sedative effects of medetomidine used in premedication seem unlikely (40, 41).

Regional anesthesia may add a crucial dimension to OFA (33, 42), with nerve blockade preventing nociceptive signal transmission to the dorsal horn of the spinal cord (43). Appropriate duration of action, promoted by precise ultrasound-guided drug deposition, also reduces central sensitization, spares postoperative opioid use, and improves long-term pain outcomes (43, 44). For these reasons, this technique has been suggested as the cornerstone of OFA in the human and veterinary literature, especially for orthopedic procedures as it provides effective intra and postoperative analgesia (42). In our study, a single fentanyl bolus was administered to 9 out of 20 dogs showing an isolated onset of nociception during arthrotomy, in contrast to one study of FS block for TPLO in dogs that describes total avoidance of opioids in the perioperative period (26). This discrepancy can be explained by a strict cutoff for rescue perioperative analgesia combined with a partial blockade of the arthrotomy and skin incision sites.

First, we set a cutoff for intraoperative rescue analgesia, as a 20% increase in MAP, HR, and/or RR, stricter than the 25–30% cutoff previously described in two similar studies evaluating FS blockade for TPLO (23, 24). This could have contributed to the higher proportion of dogs receiving rescue analgesia during surgery in our study compared to them (23, 24). However, this 20% cutoff has also been commonly used in various recent studies investigating analgesia related to femoral and sciatic blockade in stifle surgery (22, 25–28). All studies reported lower proportions of dogs receiving intraoperative opioid rescue analgesia than the results we present here, but in the majority of these studies, the surgery was limited to tibial osteotomy (22, 26, 27), or the dogs received opioids as premedication (25, 28).

Second, in our case, we can also explain these higher-than-described fentanyl requirements during the arthrotomy phase, by a partial FS block in some dogs. The inguinal approach of the femoral block, combined with sciatic nerve blockade, has been routinely described in humans and several veterinary species for analgesia of the knee (43). They target the main stifle dermatomes and sensitive innervation of the joint, provided by the articular branches of the saphenous, tibial, and fibular nerves. Both the distal end of the femur and the tibial plateau are also innervated by their ramifications, except the caudal aspect of the femoral trochlea and cutaneous area that is supplied by the obturator nerve in some animals. The cutaneous femoral nerve is also involved in sensitization of the cranial aspect of the thigh to a variable extent among the canine population. Hence, desensitization of the FS nerves via an inguinal approach may neither completely desensitize the stifle joint nor the whole skin incision during TPLO associated with arthrotomy (45). This remains the main hypothesis for the nociception phases that were successfully addressed with short-lived injections of fentanyl during arthrotomies in nine dogs of our study. The results of a recent study comparing perioperative rescue analgesia in dogs undergoing TPLO surgery and receiving a saphenous and sciatic nerve block with or without a supplementary obturator nerve block further supported our hypothesis. Indeed, of the 15 dogs receiving the obturator nerve block, only 4 needed rescue analgesia compared to 12 out of 15 in the control group (46). This is in accordance with the fact that the two dogs that received the femoral block through a pre-iliac approach in our study never needed rescue perioperative analgesia. Another explanation could be a partial block of the saphenous branch due to incomplete spread of the local anesthetic solution along the nerve, due to insufficient injection volumes ranging from 0.06 to 0.011 mL/kg in this study, which are slightly lower than what is usually reported for non-ultrasound-guided femoral block (8, 43). It remains unlikely that this accounted for the nociception during the arthrotomy phase.

Based on the theoretical 6- to 8-h duration of action of bupivacaine, we expected the blocks to fully cover the whole intraoperative and early postoperative period analgesic needs (43, 47). In addition, in agreement with reports of sensitive blockade associated with bupivacaine lasting more than 12 h in dogs (47), we observed optimal postoperative analgesia without any opioid requirement for 24 h. These observations may challenge the benefits of further extending the duration of the blockade with dexmedetomidine reported elsewhere (22, 26). All the dogs were ambulatory, bright, alert, and responsive 4 h after recovery despite partial motor blockade present for at least 6 h in all dogs. As sensitive blockade is supposed to outlast motor blockade (22, 43), we can assume that it was still ongoing at least 6 h after recovery and that residual analgesia persisted a lot longer. This is further supported by the total avoidance of rescue analgesia during the postoperative period, which in the authors’ opinion would hardly be explained by the NSAIDs and ketamine alone.

This study has several limitations linked first to its descriptive nature including the lack of a control group. If the success and feasibility of tOF-poA following OFA or OSA as defined in our study in dogs undergoing TPLO was evident, it remains difficult to establish a direct correlation between total avoidance of systemic opioids and the quality of recovery and return to normal behavior. Indeed, in this study, the adequate management of intraoperative nociception could, alone, explain the very good convalescences observed, whether systemic opioids had been added in a control group or not. Second, the two anesthetists who performed the pain scores postoperatively were aware of the treatment and objectives of the study, which can lead to subjectivity in pain assessment. In addition, two different people assessing the pain scores can introduce variability (48). However, two different pain scoring systems, each based on objective and subjective criteria, one of which specifically validated for orthopedic conditions (49) were used concomitantly in an attempt to mitigate variability. Moreover, we chose to administer postoperative rescue analgesia if at least one of the two pain scores reached the threshold value. This approach is intended to decrease the likelihood of not detecting pain due to subjectivity and variability, but could also increase the probability of administering opioids postoperatively. However, no dog in our study reached any of these scores, and their general behavior, food intake, and emunctory functions were also recorded as qualitative indicators of overall good recoveries. Acepromazine given right after osteotomy could have modified the pain evaluation. Considering the timing of administration of more than 60 min before discontinuation of isoflurane, the low dose, and the subsequent low sedation scores at 1 h, we suggest that it is unlikely to interfere in a clinically significant manner with pain scoring even if this hypothesis cannot be completely ruled out in our experimental setting.

## 5 Conclusion

This study shows that tOF-poA following OFA or OSA can be achieved in dogs presenting with TPLO, without compromising the patient’s comfort. The implementation of this protocol in our population led to very calm and uneventful recoveries. No dog showed any sign consistent with painful behavior during evaluation for the whole study period. However, further studies, such as prospective controlled trials, would strengthen the results presented in this observational study and allow us to weigh the benefits against the inconveniences of opioid-free protocols on such outcomes. This could also become crucial, as the human opioid crisis might restrict veterinary access to several opioids in the future.

## Data availability statement

The raw data supporting the conclusions of this article will be made available by the authors, without undue reservation.

## Ethics statement

The study design was approved by the University Institutional Animal Care and Use Commitee of Toulouse University (SSA_2022_013). The study was conducted in accordance with the local legislation and institutional requirements. A specific written informed consent was not obtained from the owners for the participation of their animals in this study because it is descriptive of a standard analgesia protocol at our institution, with no control group. General informed consent, including anesthesia practice, was obtained from the owners prior to surgery as is standard practice before any intervention at our institution.

## Author contributions

CD: Conceptualization, Data curation, Formal analysis, Investigation, Methodology, Project administration, Supervision, Writing – original draft, Writing – review & editing, Validation, Visualization. SF: Conceptualization, Data curation, Investigation, Methodology, Software, Writing – original draft, Validation, Visualization. MSF: Methodology, Writing – review & editing, Conceptualization, Validation, Visualization. ML: Validation, Writing – review & editing, Methodology, Visualization. SJ: Validation, Visualization, Writing – review & editing. GJ: Conceptualization, Formal analysis, Methodology, Project administration, Supervision, Validation, Visualization, Writing – original draft, Writing – review & editing.
